# Acid-functionalized PVA composite membranes for pervaporation-assisted esterification†

**DOI:** 10.1039/d4re00388h

**Published:** 2024-11-15

**Authors:** Julia Piotrowska, Christian Jordan, Kristof Stagel, Marco Annerl, Jakob Willner, Andreas Limbeck, Michael Harasek, Katharina Bica-Schröder

**Affiliations:** a Institute for Applied Synthetic Chemistry, TU Wien Getreidemarkt 9/E163 Austria katharina.schroeder@tuwien.ac.at; b Environmental and Bioscience Engineering, Institute of Chemical, TU Wien Getreidemarkt 9/E166 Austria michal.harasek@tuwien.ac.at; c Institute of Chemical Technologies and Analytics, TU Wien Getreidemarkt 9/E164 Austria

## Abstract

Composite flat-sheet membranes functionalized with imidazolium-based ionic liquids (ILs) grafted to poly(vinyl alcohol)/glutaraldehyde as a catalytic layer were developed to enhance the esterification between *n*-butanol and acetic acid. The functionalized membranes were produced *via* dip-coating commercial pervaporation membranes, and two distinct Brønsted-acidic ILs with an imidazolium-based cation and different (hydrogen sulfate [HSO_4_]^−^ or bromide [Br]^−^) anions were compared. Compact, 12 μm-thick, defect-free catalytic layers were observed on top of the pervaporation membrane supports, and the determined penetration depth of the ILs confirmed their presence in the upper part of the coating. While both ILs could significantly promote the esterification of *n*-butanol and acetic acid, the [HSO_4_]^−^ anion catalyzed the formation of butyl acetate more effectively than [Br]^−^-based species, resulting in yields of up to 50% over 15 h. Furthermore, the coated membranes exhibited enhanced water separation factors compared to the unfunctionalized one owing to the reduced swelling of the coated membranes accompanied with their diminished wettability.

## Abstract 2

Composite flat-sheet membranes functionalized with imidazolium-based ionic liquids (ILs) grafted to poly(vinyl alcohol)/glutaraldehyde as a catalytic layer were developed to enhance the esterification between *n*-butanol and acetic acid.

## Graphical abstract

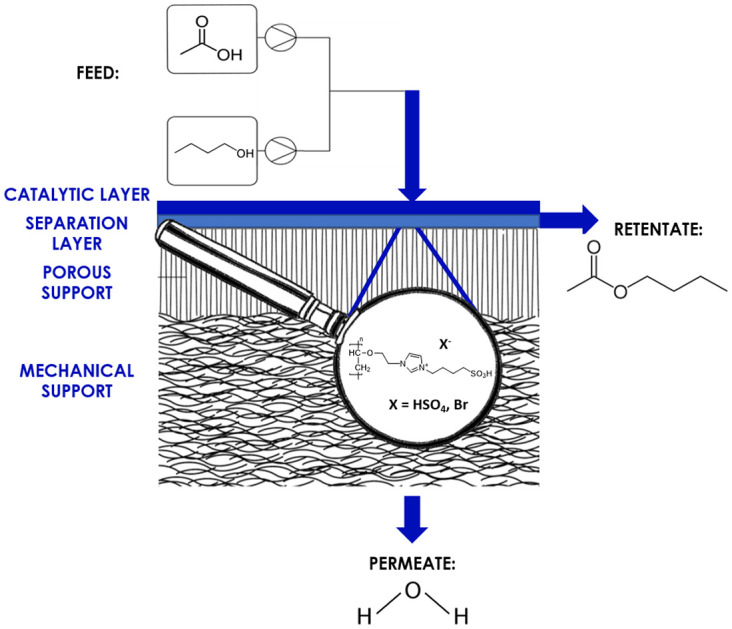

## Introduction

*n*-Butyl acetate belongs to a group of widely used and relevant intermediate chemicals, finding extensive application in the chemical and pharmaceutical industries. It may play the role of a versatile solvent with low toxicity and is used in acrylic polymers, coatings and paints. The optimization of its synthesis is still intensively investigated because of the growing demand for the replacement of hazardous, conventional solvents.^1,2^ Commonly, *n*-butyl acetate is synthesized during the esterification of *n*-butanol and acetic acid in the presence of an acidic catalyst, *e.g.* H_2_SO_4_, HF, HCl, or H_3_PO_4_.^3–5^ Esterification reactions are reversible; thus, the strategy to overcome thermodynamic constraints is crucial. One of the ways to obtain higher conversions *via* shifting the chemical equilibrium towards ester formation is based on the coupling of the reaction with constant water removal.^6–8^

Pervaporation is a membrane-based separation method applied for liquid mixtures. It is based on the partial evaporation of the feed in a dense membrane.^9^ The difference between the partial pressures of the components at the two membrane sides becomes the driving force for separation. This leads to the formation of a permeate, enriched in the component of interest. Typically, a permeate is cooled and kept under reduced pressure; thus, it can be condensed and collected in the liquid form.^10^ The sorption–diffusion model can be applied to describe the mechanism of transport through a membrane.^11^ Among the plethora of membrane materials available, poly(vinyl-alcohol) (PVA)-based membranes exhibit outstanding permselectivity and are state-of-the-art hydrophilic membranes for dehydration.^12^ Functionalization of a membrane with a catalyst offers an excellent strategy to combine separation with catalysis in so-called pervaporation-assisted esterification. The formed water is selectively removed as the reaction proceeds; thus, thermodynamic equilibrium can be broken and higher conversions can be achieved.^6^ Moreover, for membranes modified with a catalyst, the major drawbacks associated with conventional, homogeneous catalysis, such as challenging catalyst separation and recovery, can be overcome.^13–15^ The favoured configuration of a catalytically active membrane is a multi-layer composite, where the catalyst is embedded in a medium on top of a membrane selective layer. This structure allows for the independent tailoring and optimization of both separation and catalytic properties.^16^

Functionalized ionic liquids (ILs), and especially Brønsted-acidic ILs, have been successfully applied for esterification reactions as versatile catalysts.^16–18^ They are favoured due to their remarkably low volatility and vapour pressure, as well as high thermal stability and low toxicity.^19,20^ Furthermore, ILs were also found to positively influence the pervaporation process.^21,22^ The catalytic activity of ILs can be combined together with their favourable impact on water removal by their immobilization onto pervaporation membranes. Although several strategies for the incorporation of ILs to the structure of membranes have been proposed,^23–26^ their catalytic activity in functionalized membranes has not been widely investigated yet. However, an interesting approach was presented in the work of Zhang *et al.*,^27^ where an imidazolium-based IL containing a [HSO_4_]^−^ anion was immobilized onto the surface of a PVA membrane for the esterification reaction between ethanol and acetic acid.

Herein, we report a membrane coating method, based on the procedure described by Zhang, which was adjusted for the purpose of using commercially available high-performance pervaporation membranes as composite supports. Two different Brønsted-acidic ILs with a functionalized imidazolium-based cation and different anions (and thus acidity) were grafted in PVA-based solution and coated on the membrane's top surface. The influence of the immobilized ILs on pervaporation was compared, and the catalytic activity of the PVA/IL-coated membranes was investigated during the esterification reaction between *n*-butanol and acetic acid.

## Experimental section

### Materials

For the preparation of the coated composites, commercial PVA-based pervaporation membranes, type PERVAP™ 4100 (standard membrane for the dehydration of volatile organic mixtures), were purchased from DeltaMem AG. The technical data sheets of the membranes are given in the ESI† (Table S1). The membranes were coated using polyvinyl alcohol, 98–99 wt% pure hydrolysed, medium molecular weight (average M.W. 57 000–66 000), from abcr GmbH, glutaric dialdehyde, 25 wt/vol% solution in water from Thermo Scientific Acros, potassium persulfate, ≥99.0 wt% pure from Sigma Aldrich, deionized water, and the ionic liquids.

**Scheme 1:**
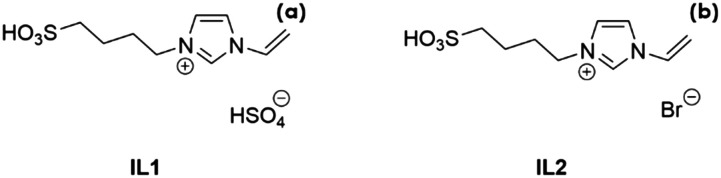
Molecular structures of IL1 (a) and IL2 (b).

Two different ILs were used: 3-(4-sulfonyl)-1-vinyl-imidazolium hydrogen sulfate, henceforth referred to as IL1, and 3-(4-sulfonyl)-1-vinyl-imidazolium bromide, IL2 (Scheme 1). The ILs were synthesized according to the literature^28^ (for the details, see the ESI).

For the pervaporation tests, acetic acid puriss. analytical sp and butyl acetate, HPLC grade, 99.7 vol%, were purchased from Sigma Aldrich, while *n*-butanol, 99.9 vol%, was purchased from Honeywell. For the two-component KF titration, HYDRANAL-Solvent E and HYDRANAL-Titrant 5 E were obtained from Honeywell Fluka. As the co-solvent for the permeate components for the gas chromatography measurements 1,4-dioxane EMPLURA® was obtained from Sigma Aldrich.

### Preparation of the composite membranes

The catalytic coating layer was prepared by dissolving 5 g of PVA in 95 g of distilled water with stirring for 24 h at 100 °C to form a homogeneous PVA solution. Afterwards, 2 g (40 wt% of PVA mass) of the IL was added, together with 0.03 g of polyphenylene sulphide (1.5 wt% of the IL mass) to initiate the graft polymerization reaction. The mixture was stirred vigorously for 12 h, at 75 °C, under a nitrogen atmosphere. After the reaction had finished, the mixture was cooled down to room temperature and left standing for 3 h. For the distribution of the IL/PVA mixture over the membrane top surface, a dip-coating method was applied. The membrane support was cut to a square shape sized 6 cm × 6 cm. Directly before the coating, 1 ml of 25 wt/vol% glutaric dialdehyde aqueous solution (corresponding to a PVA : GA 5 : 1 w/v ratio) was added to the PVA/IL mixture and stirred for 1 min. Subsequently, the solution was poured into a Petri dish and the top surface of a cut membrane was immersed in the solution as the gelation process proceeded. After 10 min, a gel-like coating on the membrane surface formed on the membrane top surface. The coated membranes were then carefully withdrawn from the solution, transferred to a vacuum oven, and dried at 60 °C for at least 48 h for complete water removal.

### Characterization of the coating solutions and coated composites

All the coating solutions (pure PVA and PVA/IL1 or IL2) were tested by ^1^H NMR to verify the grafting reaction. Samples (1 ml of each solution) were precipitated and washed with ethanol for complete water removal, followed by drying in a high vacuum. The masses of the precipitates were measured. NMR spectra were recorded with D_2_O solutions using a Bruker Avance Ultra-Shield 200 MHz NMR instrument.

Based on the mass of the precipitate, the degree of grafting (DG%) was calculated using eqn (1):1
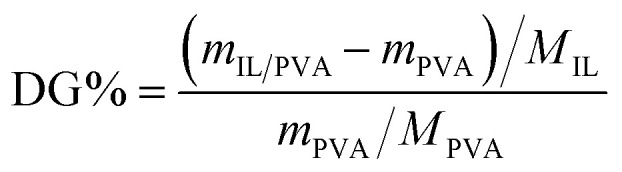
where *m*_IL/PVA_ and *m*_PVA_ stand for the masses of the dried IL-grafted PVA and pure PVA precipitates, respectively, and *M*_IL_ and *M*_PVA_ are the molar masses of the ionic liquids and PVA molecular weight. For *M*_PVA_, the molar mass of the PVA monomer was considered (44.05 [g mol^−1^]).

Fourier transform infrared spectra (FT-IR) of the coating solutions and membranes' top surfaces were obtained by FT-IR on a VERTEX 70 instrument (Bruker Optics) under ambient conditions in the range of 4000–1000 cm^−1^ using an attenuated total reflection method (ATR).

The morphology of the coated membranes was investigated by scanning electron microscopy (SEM) (EM-30, COXEM). Cross-sections of the composites were obtained by freezing in liquid nitrogen, followed by their fracturing. Due to the non-conductive nature of the samples, the membranes were sputtered with a thin layer of gold by an ion sputter coater (SPT-20, COXEM).

LA-ICP-MS was used to investigate the penetration depth of the ILs. LA-ICP-MS measurements were carried out with the laser ablation system imageGEO193 from Elemental Scientific Lasers (Bozeman, MT, USA) coupled with an iCAP Q ICP-MS system from ThermoFisher Scientific (Bremen, Germany). The samples were irradiated by a laser and measured under the following conditions: helium flowrate: 800 ml min^−1^, line scan (2 mm) spot size: 30 × 30 μm, fluence: 1.5 J cm^−2^, scan speed: 250–25 μm s^−1^, repetition rate: 50 Hz, overlap: 25 μm. The mass spectrometer parameters were as follows: plasma power: 1550 W, argon flow rates: 13.8 l min^−1^ (cooling gas), 0.85 l min^−1^ (nebulizer gas), 0.8 l min^−1^ (auxiliary gas), dwell time: 10 μs. The isotopes measured for the determination of the IL's presence were as follows: ^13^C, ^34^S (for IL1) and ^13^C, ^79^Br (IL2). Data were evaluated using Iolite software.^29^

The wetting properties of the uncoated and coated membranes were investigated *via* static water contact angle (CA) measurements, carried out with a DSA30 Drop Shape Analyzer (KRÜSS, Germany). The top surfaces of the membranes were measured with a sessile 4 μl droplet of deionized water, at 22 °C. Each membrane was measured 5 times at different spots. Based on the images, using the Young–Laplace fitting model, the average contact angle values were calculated.

The acid capacities of the uncoated and IL/PVA-coated membranes were compared by acid-based titration. The samples were cut into 5 cm^2^ pieces and kept in 25 ml of 0.05 M NaOH solution for 24 h, under stirring conditions at 80 °C. Afterwards, the solutions were titrated with 0.05 M HCl solution, using phenolphthalein as the indicator. For the acid capacity AC [mol g^−1^] calculation, eqn (2)^27^ was applied:2
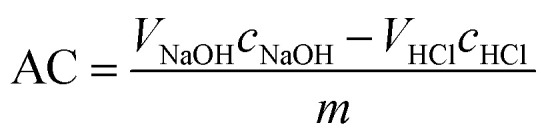
where *V*_NaOH_ and *c*_NaOH_ stand for the volume of the NaOH sample (10 ml for each titration) and known concentration of NaOH (0.05 M) respectively, *V*_HCl_ is the volume of the titrant consumed to neutralize NaOH, *c*_HCl_ is the known concentration of the titrant HCl (0.05 M), and *m* is the mass of the membrane.

The mass swelling degree (MSD) was determined with the following procedure: small pieces (2 cm × 2 cm) of uncoated, neat-PVA-coated and PVA/ILs-coated membranes were dried at 55 °C in a vacuum oven for 24 h. The mass of each dried membrane (*M*_0_) was determined and the membranes were transferred to the solution with the same chemical composition as for the subsequent pervaporation tests (80 wt% *n*-butanol + 10 wt% *n*-butyl acetate + 5 wt% acetic acid + 5 wt% water). The mixture and membranes were heated up to 80 °C. After 24 h, the mixture was cooled down, and the membranes were withdrawn from the solution. After the removal of superfluous liquid, the mass of each wet membrane was measured (*M*_1_). The MSD [%] was calculated using eqn (3):3
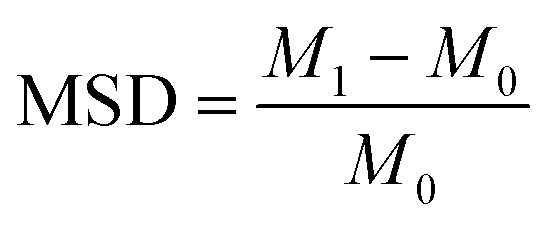


### Pervaporation experiments for assessing the water separation

**Fig. 1:**
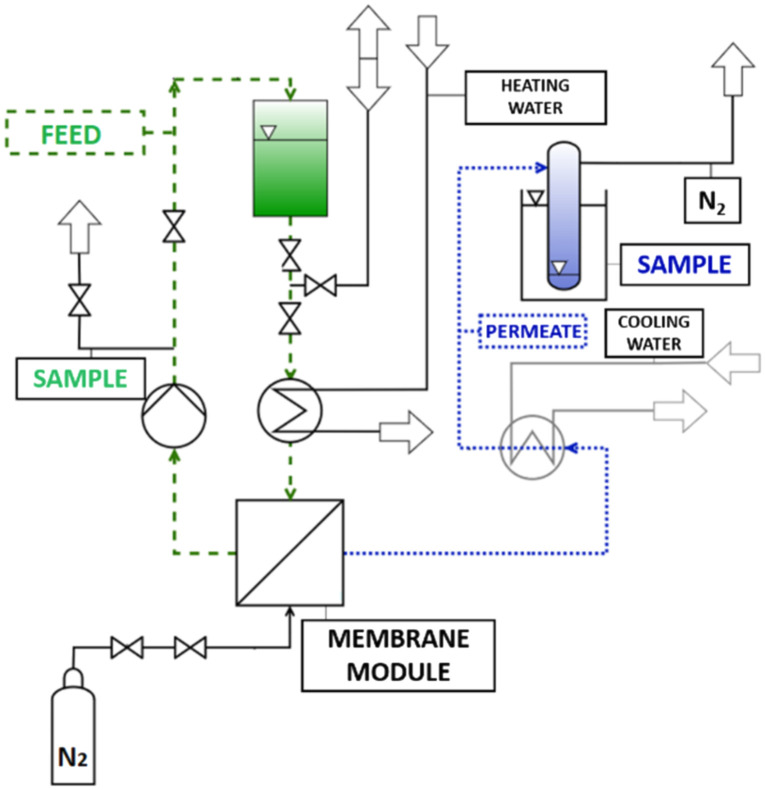
Schematic representation of the pervaporation system, adopted from Annerl.^30^

The ability of the membrane to remove water was tested by pervaporation tests, carried out with the set-up presented in Fig. 1, adapted from Annerl.^30^ The pervaporation apparatus consisted of a stainless-steel feed tank, pump, an external heating bath, equipped with silicon tube wound around a stainless-steel, circular-shaped membrane module with an effective membrane surface area of 67.93 cm^2^. Before the experiment, the membrane was cut to fit the round module (*d* = 9 cm^2^) and conditioned in the test solution for 24 h at 4 °C.^31^ One litre of the separation mixture was fed to the tank. The pervaporation system was operated under the following conditions: the heating bath was set to 95 °C, yielding a temperature of 90 °C inside the module. The membrane was flushed with nitrogen (used as a sweep gas) at a pressure set to 3 and 2 bar at the feed and permeate side, respectively. The formed vapour permeate condensed in the vials (kept in a cooling trap), whereas the liquid retentate was circulated back to the tank. The whole experiment lasted 8 h, and the permeate was collected in 2 h intervals, whereas the feed/retentate sample was collected directly from the tank each 30 min.

The water content of the permeate was measured *via* Karl Fischer titration (KF) using an Eco KF Titrator from Metrohm. The samples with a chemical composition located on the phase diagram in the miscibility gap^32,33^ were dissolved in 1,4-dioxane co-solvent. The remaining permeate components were determined by gas chromatography (GC) using a Shimadzu GC-2010 Plus instrument, equipped with a Restek™ RTX-Volatiles capillary column (serial number: 541046, length of 60 m, inner diameter of 0.53 mm, and film thickness of 2 μm). The following heating programme was applied: 25–140 °C (10 °C min^−1^ heating rate), 140–200 °C (35 °C min^−1^), 2 min isotherm.

The collected feed was tested by KF and by FT-IR in the range of 4000–400 cm^−1^. The wt% of the feed components was determined using the partial least-squares regression technique, together with MATLAB solver.^34^ After the calibration based on the FT-IR spectra, MATLAB script, developed by Annerl,^30^ was applied to calculate the exact feed composition.

### Calculations

To evaluate the membrane separation performance, two main parameters were considered: the membrane flux *J*, considering the partial flux of component *i*, and separation factor *α*_*i*_,^11,27^ which can be described with eqn (S1)–(S3); and the enrichment factor *β*_*i*_, calculated from eqn (S4),† which indicates the degree to which component *i* (of greater permeability) is enriched. All the equations can be found in the ESI.†

### Catalytic activity tests – batch mode and esterification-coupled pervaporation

The catalytic activities of the neat ILs and the PVA/IL-coated membranes were tested in three reaction systems: homogeneous, with 15 mg of catalyst uniformly distributed in the reaction media; heterogeneous, with the same mass of catalyst immobilized on small pieces (2 × 2 mm) of the coated membranes; and a control system, in the absence of any catalyst. The reactions were carried out in 8 ml flasks for 24 h at 90 °C, under magnetic stirring. The reaction media consisted of *n*-butanol (3364 μl) and acetic acid (57 μl), which corresponded to a 40 : 1 molar ratio (*n*-butanol was used in excess as a solvent). At set intervals, 47 μl of the reaction media was collected, specifically at 0, 1, 1.5, 2, 3, 4, 6, 24 h. After the addition of the internal standard (dodecane), the samples were analysed by calibrated GC.

After 24 h, the supernatant of both heterogeneous systems was collected and *n*-butanol was evaporated. The precipitates were dissolved in deuterated methanol. ^1^H NMR spectroscopy with 3,5-bis(trifluoromethyl)benzoic acid as the internal standard was used to determine the leaching of the IL, with a limit of detection of 0.05 wt%.

Simultaneous catalysis and water removal were studied during esterification-coupled pervaporation, in the same reactor as for the pervaporation, run under the same conditions, with a feed composition of 760 ml of *n*-butanol and 40 ml of acetic acid. The experiments were carried out for the functionalized membranes and the uncoated membranes, with the catalyst dissolved in the reaction mixture.

## Results and discussion

### Establishing the coating procedure

The first challenge to be addressed was establishing the detailed and reproducible procedure for coating the membrane supports. To the best of our knowledge, no investigation of PVA/IL layer immobilization on top of a commercial pervaporation membrane support has yet been reported. The crucial aspect of the coating technique was to ensure that the coated PVA/IL layer was permanently immobilized on the surface of the membrane and would not be washed away under the harsh esterification conditions.

**Scheme 2:**
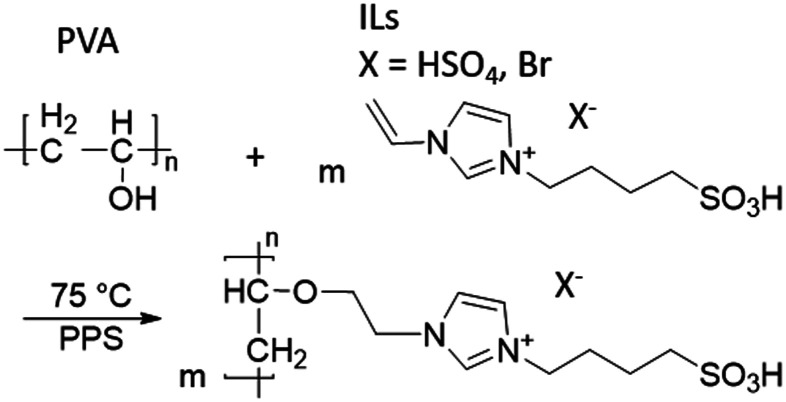
Grafting between IL1/IL2 and PVA.

Prior to coating the membranes, it was necessary to confirm the completion of grafting of the ILs on to PVA *via*^1^H NMR spectroscopy. Both spectra of the IL/PVA solutions exhibited additional signals for the ILs characteristic groups when compared to the spectrum of pure PVA, located in the range of 10–5.5 ppm (see ESI,† Fig. S1), together with the assignment of the characteristic signals of ILs. The degree of grafting (DG%) was calculated based on the masses of PVA and the IL-modified PVA dried precipitates, with the DG% determined as 3.22 and 1.31 for IL1 and IL2, respectively, which confirmed the successful grafting of the ILs to PVA. The possible polymerization mechanism, as postulated also by Zhang *et al.*,^27^ is depicted in Scheme 2.

**Fig. 2:**
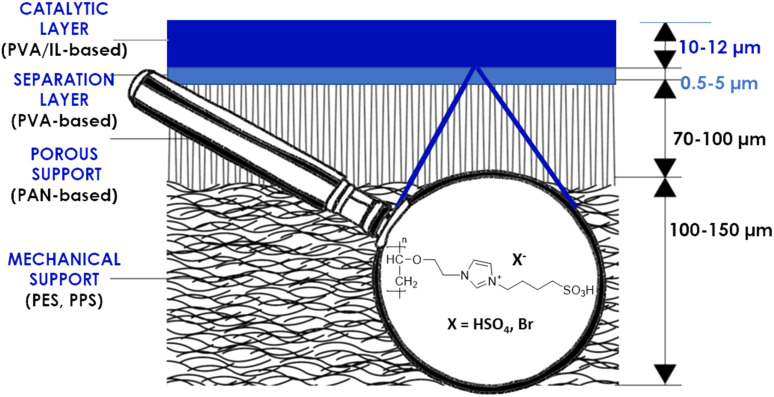
Schematic representation of the coated membrane.

In the first coating attempts, the PVA/IL-grafted solution was simply dip-coated on the membrane's surface and dried in a vacuum oven. This resulted in poor stability and an insufficient coating thickness, confirmed *via* its absence in the SEM images. As described in the literature,^35^ the addition of a cross-linking agent, such as glutaraldehyde (GA), can play a crucial role in the formation of stable PVA-based coatings. Adjustment of the GA/PVA ratio and gelation time had a significant impact on the morphology of the obtained coating. Exceeding the exact gelation time or GA/PVA ratio yielded membranes with a non-uniform coating, whereas for too short gelation times, no coating was formed. Eventually, a gelation time of 10 min and PVA : GA 5 : 1 w/v ratio were identified as the optimum conditions, and allowed observation of the macroscopic formation of stable, thin coatings on the membranes' top surfaces. The multilayer composites (Fig. 2) consisted of the membrane support, namely the commercially available PVA-based pervaporation membrane, and the self-synthesized catalytic top layer, which contained one of the two investigated ILs, immobilized in PVA.

### Characterization of the coated composites

**Fig. 3:**
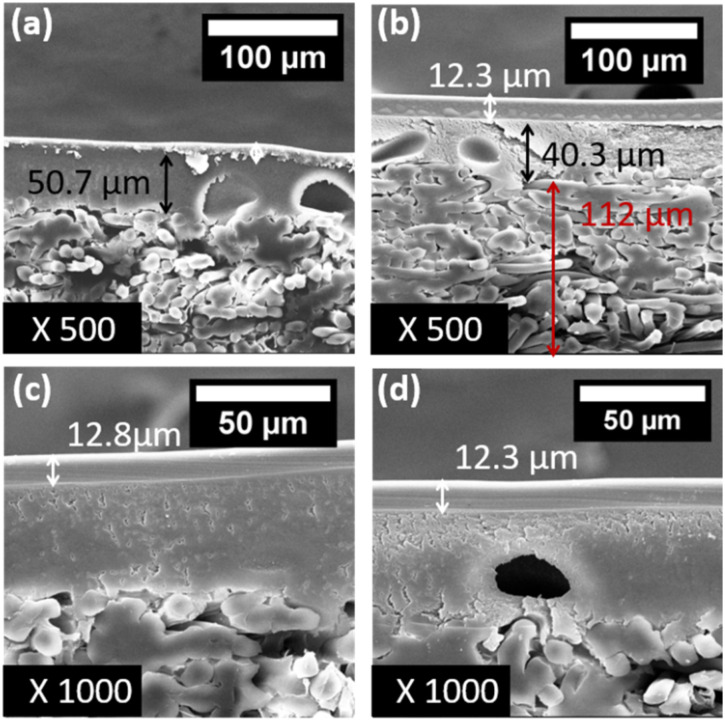
SEM images at 500× magnification: uncoated membrane (a) and membrane coated with PVA/IL1 (b). SEM images at 1000× magnification: membrane coated with PVA/IL1 (c) and membrane coated with PVA/IL2 (d).

The membranes' cross-sections were visualized *via* SEM and the thicknesses of all the observed layers were determined. As visible in Fig. 3, the untreated commercial membranes exhibited the presence of a dense, top separation layer (a). For the functionalized membranes (b–d), the formation of 12.8 and 12.3 μm thick catalytic layers was observed for the PVA/IL1- and PVA/IL2-coated membranes, respectively, without the formation of macrovoids and high homogeneity and proper adherence to the separation layers. The morphology of the ∼40 μm thick porous PAN support, observed under SEM, was not affected by the PVA/IL coating, according to the SEM images. Moreover, the fibre-like, polyester-based mechanical support was observed under SEM and exhibited macro-voids for both the PVA/IL-coated and uncoated membranes. The catalytic (self-coated) and separation (manufactured) PVA-based layers merged and it was not possible to distinguish them visually; therefore, further characterization of the top coating was necessary.

**Fig. 4:**
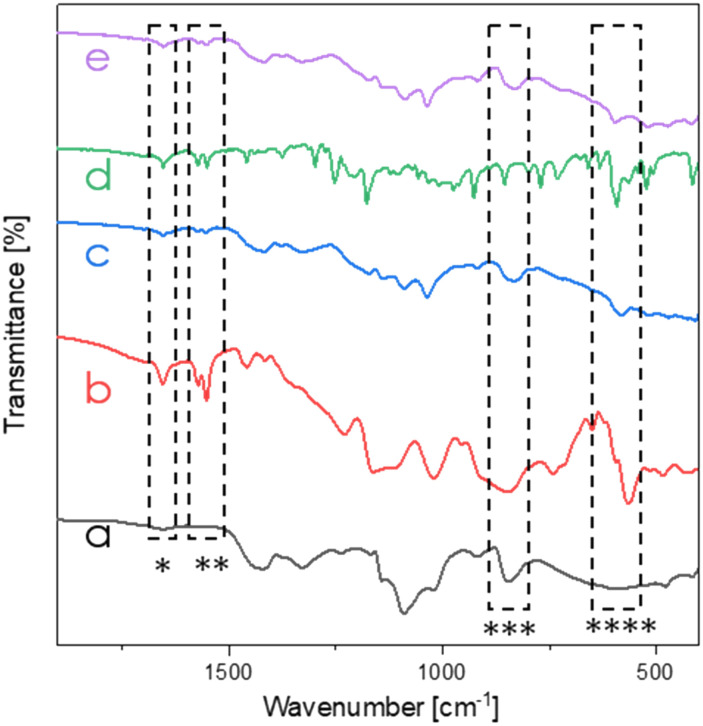
FT-IR membrane spectra (range 2000–450 cm^−1^) of the uncoated membrane (a), neat IL1 (b), PVA/IL1-coated membrane (c), neat IL2 (d) and PVA/IL2-coated membrane (e).

FT-IR measurements were carried out both for neat ILs, as well as for the top membrane surfaces of the uncoated and PVA/IL-coated samples to confirm the presence of immobilized ILs on the top surfaces of the membranes. As visible in Fig. 4, comparison of the spectra of the uncoated and PVA/IL-coated membranes revealed the appearance of additional peaks that could be attributed to the ILs. Specifically, sharp peaks at 1650 cm^−1^ (*) and 1550 cm^−1^ (**) were assigned to the stretch vibrations of C=C and C=N double bonds in imidazole ring, respectively, while the peaks at 830 cm^−1^ (***) and 640 cm^−1^ (****) were attributed to torsion of the imidazole ring.^36^

**Fig. 5:**
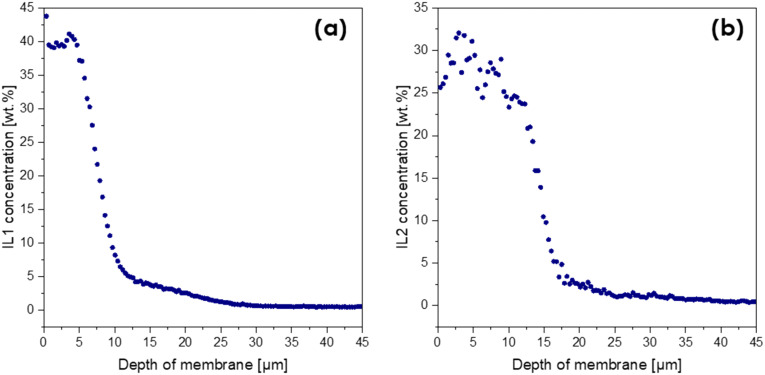
Weight fractions of IL1 (a) and IL2 (b) as a function of membrane depth determined using the LA-ICP-MS method.

Apart from the confirmation of the ILs' presence in the coated layer, additional information about their penetration depth into the layered-composite was required. Laser ablation inductively coupled plasma mass spectrometry (LA-ICP-MS) was applied as it can provide quantitative information about the vertical distribution of selected elements in the top layers of the membranes and was thus ideally suited to address this issue. This technique yields spatially resolved elemental information about solid samples. Focused laser pulses ablate material from the sample, which is then transported by a carrier gas into an ICP for atomization and ionization. The ions are detected by a mass spectrometer. This method allows for elemental distribution imaging with <10 μm resolution or depth profiles with sub-μm resolution through repeated laser pulses at the same location. The isotopes chosen for the determination of the IL content were the isotopes of the characteristic elements included in the structures of the IL anions, namely ^13^C, ^34^S (for IL1) and ^13^C, ^79^Br (IL2).^37^ A series of standards with known IL concentrations (wt%) were measured to obtain a calibration curve, with the help of which quantitative results could be obtained for the coated membranes. Fig. 5 shows the resulting plots of the IL concentration plotted against the corresponding ablation depth, representing the depth profile.

As can be seen in Fig. 5, the concentration of IL1 reached the desired 40 wt% concentration down to a depth of 7 μm. This proves that IL1 stayed in the top part of the coating and did not migrate down to the membrane support. Thus, it should enable surface contact between the IL and reagents during esterification. For the membrane coated with IL2, the IL content at the surface was ∼30 wt%, suggesting that some amounts of IL2 penetrated deeper into the membrane support (down to ∼15 μm), which therefore would potentially decrease the catalytic activity of the membrane.

### Membrane performance for pervaporation and esterification

The composition of the mixture to be separated was chosen according to the one expected at reaction equilibrium, with the assumption that *n*-butanol plays the role of a solvent and is added in the volume *n*-butanol : acetic acid ratio of 5 : 1. The test mixture composition was as follows: 80 wt% *n*-butanol + 10 wt% *n*-butyl acetate + 5 wt% acetic acid + 5 wt% water. The water-removal ability of the membranes was determined during comparative pervaporation experiments, carried out for both the uncoated membrane and the membranes functionalized with IL1 or IL2.

**Fig. 6:**
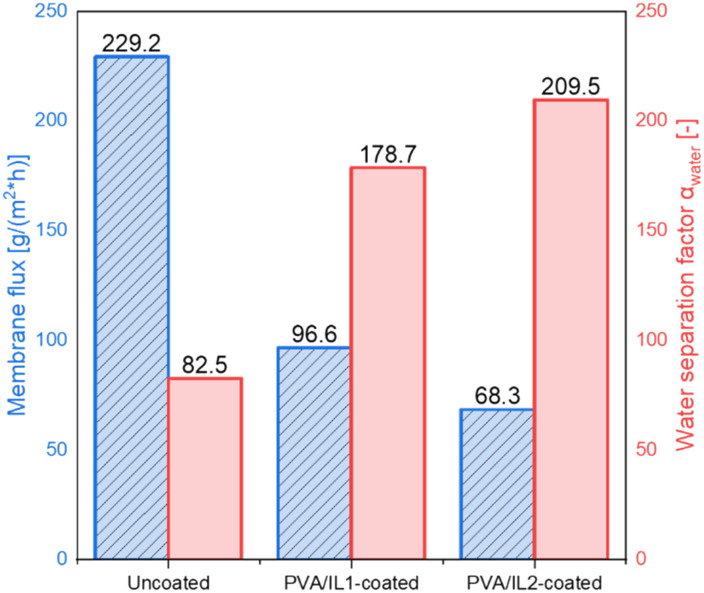
Membrane fluxes (blue, dashed) and water separation factors (red, smooth) measured for the uncoated and PVA/IL1- and PVA/IL2-coated membranes determined during pervaporation at *T* = 95 °C, sweep gas *p* = 3/2 bar (feed/permeate).

The influence of the ILs' addition on the membrane performance was evaluated by comparing their fluxes and separation factors. The results for the uncoated and PVA/IL1- or IL2-coated membranes are presented in Fig. 6. Although the flux of the uncoated membrane was the highest, reaching 229.2 g m^−2^ h^−1^, it exhibited the lowest separation factor (82.5 [−]), according to the flux–separation trade-off dependency.^38^ The highest separation performance was observed for the membrane coated with PVA/IL2, for which the flux reached 68.3 g m^−2^ h^−1^ and the water separation factor *α*_H_2_O_ was 209.5 [−]. The observed enhancement of water separation, associated with the addition of imidazolium-based ILs, was expected and supported by the literature.^39,40^ The flux and separation of membranes depend on the swelling of the selective layer due to the interaction with the liquid.^41^

**Fig. 7:**
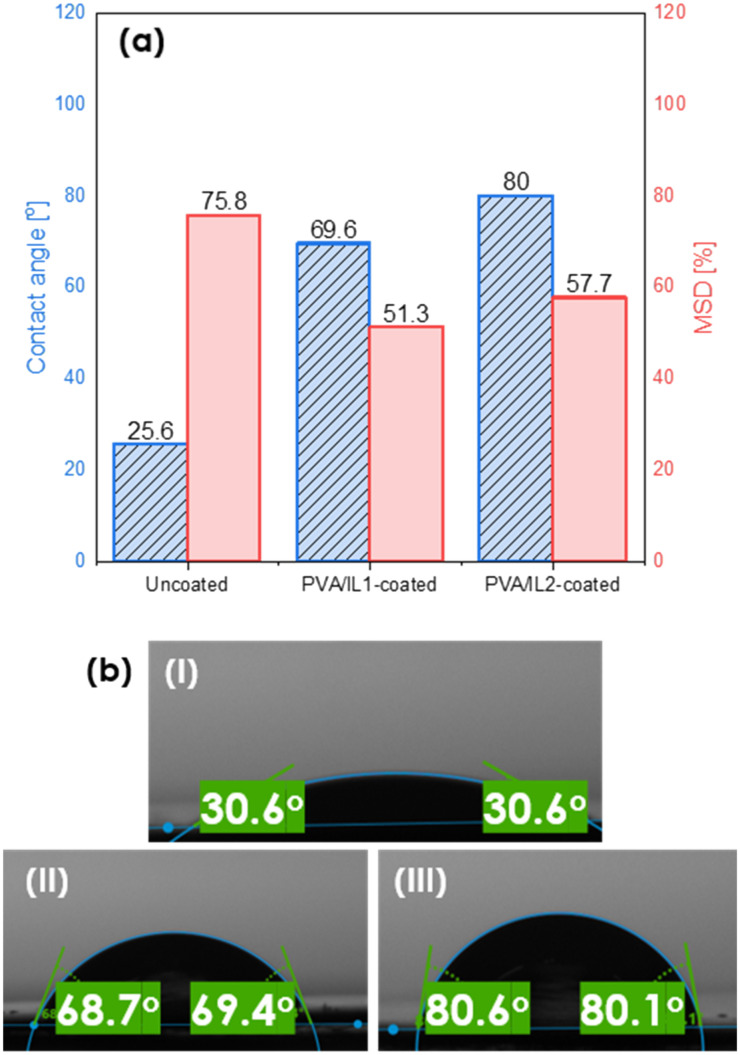
Contact angle values (blue, dashed) and mass swelling degree (red, smooth) (a) and images of membrane surfaces taken during sessile drop tests for the uncoated (I) and PVA/IL1- (II) and PVA/IL2-coated (III) membranes (b).

As can be seen in Fig. 7(a), the mass swelling degree (MSD) turned out to be generally lower for the IL-functionalized membranes, indicating the addition of ILs inhibited swelling of the membranes, resulting also in a lower membrane flux. However, it positively influenced the membranes' separation performance. The addition of ILs enhanced their water removal ability. This effect was more pronounced for IL2, which was also less hydrophilic than IL1.^42^ These findings were in compliance with the results of the contact angle (CA) measurements (presented in Fig. 7(a) and (b)). The CA was the lowest for the uncoated membrane, indicating its high hydrophilicity.

The exact composition of the permeate, determined by the combination of Karl Fisher (KF) titration and gas chromatography (GC), showed that the quantities of *n*-butyl acetate and acetic acid in the permeate samples were below the limit of detection (<0.02 and <0.03 wt%), indicating that the membranes could selectively remove water, while the other reactants were retained in the tank. As can be seen in Table 1, the most selective sample (coated with PVA/IL2) permeate had a nearly 19 times higher water content (92.9 wt%) than the feed (5 wt%), whereas for the uncoated membrane, the water enrichment factor was only 16.2 [−].

**Table 1:** Composition of the permeate determined using KF and GC

Membrane	Uncoated	PVA/IL1-coated	PVA/IL2-coated
Component			
Butanol weight fraction [wt%]	15.7	8.2	7.1
Water weight fraction [wt%]	84.3	91.8	92.9
Water enrichment factor *β*_H_2_O_ [−]	16.2	18.3	18.6

**Fig. 8:**
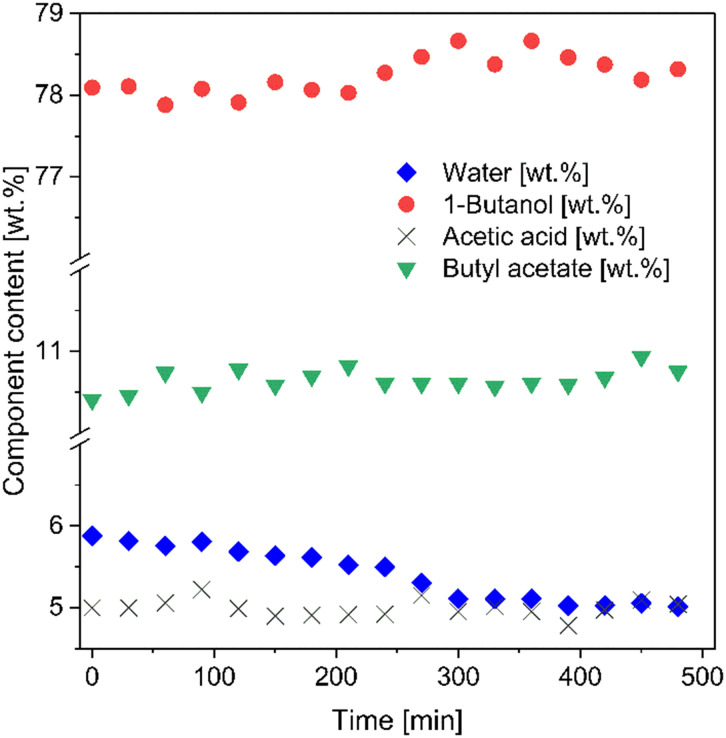
Time evolution of the weight content [wt%] of each component of the feed mixture, for the membrane coated with PVA/IL2 determined during esterification-coupled pervaporation at *T* = 95 °C, sweep gas *p* = 3/2 bar (feed/permeate).

As depicted in Fig. 8, the successful removal of water was additionally confirmed by analysis of feed the composition, as monitored *via* FT-IR at 30 min intervals. The weight fraction of water decreased from 5.9 to 5.0 after 8 h, whereas the wt% values of the other components circulating in the permeation tank slightly rose. Although the change was not significant, due to the relatively large feed-to-permeate volume ratio (1000 ml of feed to 5 ml of permeate), the observed tendency further proved that the feed components were retained and only water could pass through the membrane.

The sufficient chemical and mechanical stability of the coated membranes were confirmed based on their mass difference before and after the pervaporation tests, followed by their drying. The negligible change of mass indicated that the coating was not washed away. Moreover, FT-IR spectra (see the ESI,† Fig. S2) of the coated membranes measured before and after the pervaporation tests exhibited identical patterns. This confirmed that the formation of dissolution products or the removal of ILs did not occur, suggesting that the formed composite membranes exhibited sufficient stability under the harsh pervaporation/esterification conditions.

**Fig. 9:**
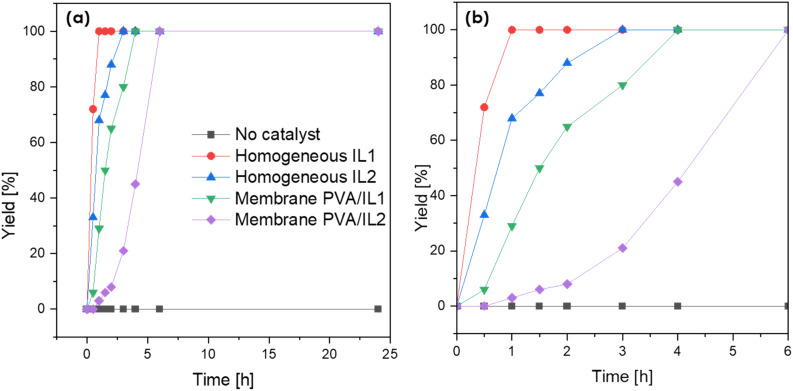
Time evolution of esterification yield measured after batch esterification (*T* = 90 °C, under stirring) with no catalyst, catalyst added directly to the reaction flask and with PVA/IL-coated membranes (a) and magnification after cut-off at 6 h (b).

Prior to the actual esterification tests in the membrane reactor, the catalytic activity of both neat ILs, as well as the PVA/IL-coated membranes, was evaluated in batch mode experiments. As visible in Fig. 9, without the addition of the catalyst, the reaction could not proceed with a rate sufficient enough to observe any conversion after 25 h. For both ILs, full conversion was achieved quicker than when they were immobilized on the membrane. This was expected due to the lack of mass-transfer limitations in homogeneous catalysis. Complete conversion was obtained after 0.5 and 3 h for IL1 and IL2, respectively. For the coated membranes, full conversion was observed after 4 h (PVA/IL1) and 6 h (PVA/IL2). The comparison between the two types of ILs indicated the superior performance of IL1 due to the high acidity of [HSO_4_]^−^.^43,44^ The higher acidity of the PVA/IL1-coated membrane was further supported *via* acid capacity (AC) measurements, with AC values equal to 0.5, 0.8, and 0.6 [mmol g^−1^] for the uncoated, and PVA/IL1- and PVA/IL2-coated membranes, respectively.

Eventually, the catalyst leaching from the coated membranes was investigated *via*^1^H NMR analysis of the supernatant (see the ESI,† Fig. S3). The absence of signals from IL1 provided evidence of the lack of IL1 in the liquid phase, and thus its stable immobilization in the catalytic layer. For the PVA/IL2-coated membrane, the signals from IL2 in the ^1^H NMR spectra revealed that 26% of the IL2 mass had migrated from the catalytic layer.

**Fig. 10:**
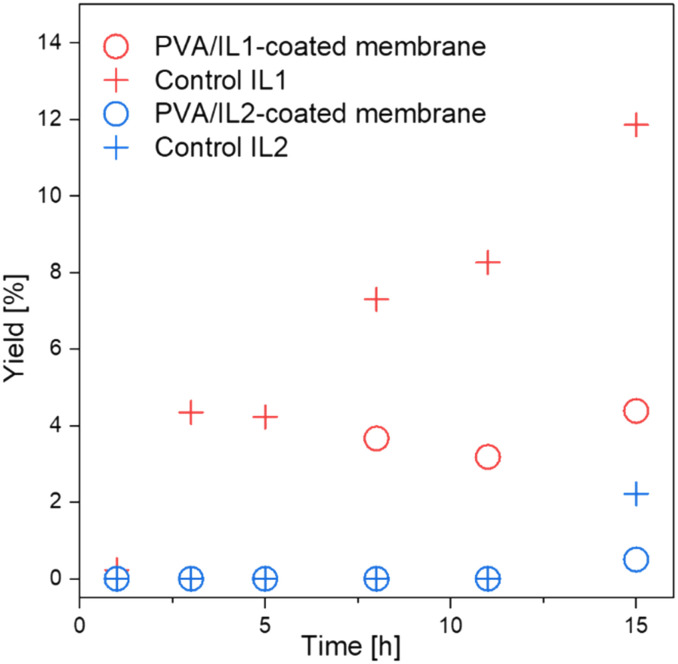
Esterification comparison between PVA/IL1- and PVA/IL2-coated membranes and control experiments (uncoated membrane, catalyst distributed in the reaction mixture) at *T* = 95 °C, sweep gas *p* = 3/2 bar (feed/permeate).

The catalytic activity and simultaneous water removal capability of the PVA/IL-coated membranes were studied together during esterification-coupled pervaporation in the membrane reactor. Experiments were conducted in the same device as for the pervaporation, with a feed composition of 760 ml of *n*-butanol and 40 ml of acetic acid (15 : 1 molar ratio). Esterification was run for 15 h under the same conditions as for the pervaporation. Two types of experiments were carried out: a test reaction with the PVA/IL-coated membranes and, for the comparison, homogeneous catalysis with a catalyst dissolved in the feed assisted by pervaporation with the uncoated membrane. The amount of dissolved catalyst was equal to the calculated mass of the IL immobilized on the surface of the membrane (based on the mass difference of the coated and uncoated membrane). As can be seen in Fig. 10, the comparison between the two ILs showed clearly the superior catalytic activity of IL1 over IL2, which was in accordance with the results from the batch esterification experiments. This ultimately confirmed the better catalytic performance of the [HSO_4_]^−^-based IL. However, the yields remained below <10% within 15 h and were in the same range as when the catalyst was dissolved in the reaction mixture during the control experiments. This indicates that pervaporation could merely compensate for the immobilization of the catalyst on the surface in comparison to the homogeneous control experiment. However, the catalyst immobilized on surface stayed on the membranes without any trace of leaching and could be potentially reused for the further esterification without requiring additional, energy-consuming separation steps.^45^

The weak performance in all the esterification-coupled pervaporation experiments can be explained by the large reactor volume compared to the relatively small membrane surface area. In this reactor configuration, a minimum feed volume of 1000 ml was required, whereas the membrane module size was relatively small (67.93 cm^2^). The mass of catalyst coated on the support was in the range of 30–40 mg. One of the strategies to increase the conversion would be to perform the esterification in a more concentrated solution; for example, with an *n*-butanol/acetic acid ratio of 2 : 1. However, in such a highly acidic and corrosive environment, the membranes could deteriorate. Moreover, some parts of the membrane reactor could be affected.

As a compromise, experiments were conducted with a lower *n*-butanol to acetic acid molar ratio (9 : 1) in the feed with the more catalytically active membrane (PVA/IL1-coated). Additionally, a control test was conducted, with the same starting reaction mixture composition (*n*-butanol : acetic acid = 9 : 1), under the same conditions, but with the uncoated membrane and in the absence of the catalyst.

**Fig. 11:**
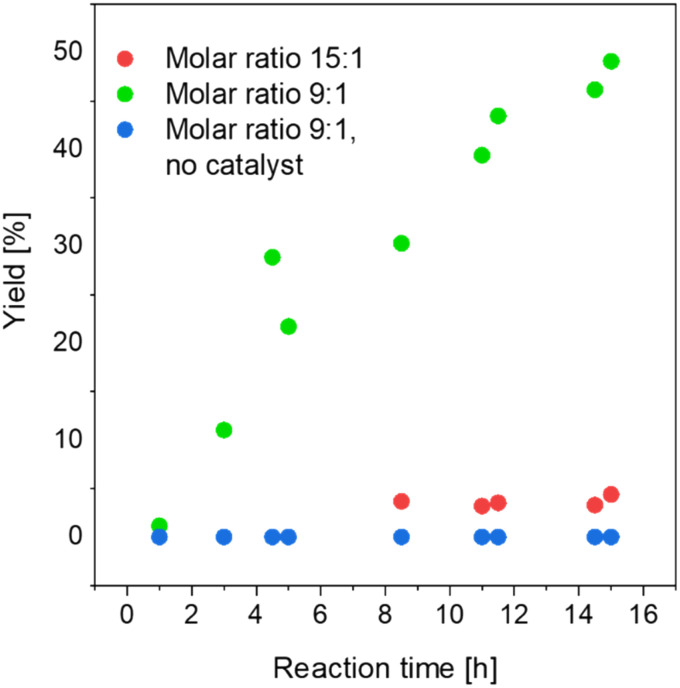
Esterification-coupled pervaporation (*T* = 95 °C, sweep gas *p* = 3/2 bar (feed/permeate)) with the PVA/IL1-coated membrane and comparisons between different *n*-butanol/acetic acid molar ratios in the feed.

As can be seen in Fig. 11, a significant increase in the conversion rate was observed in the more concentrated system. After 15 h of experiment, a 50% *n*-butyl acetate yield was reported, whereas no *n*-butyl acetate formation was observed without the catalyst after 15 h under otherwise similar conditions. The improvement of the yield in the studied time was a promising result and, as an outlook, long-term studies are proposed to build on these findings.

**Fig. 12:**
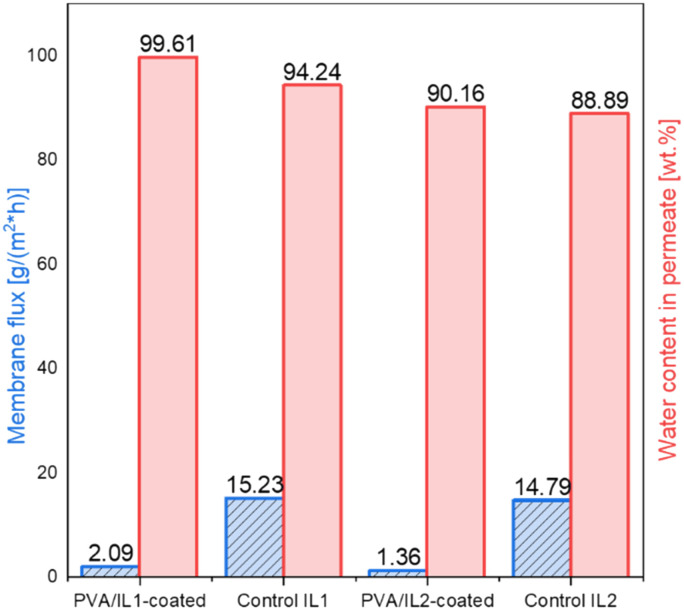
Fluxes of membranes (left, blue dashed) and water content of the permeate (right, red) obtained during pervaporation coupled with esterification experiments (*T* = 95 °C, sweep gas *p* = 3/2 bar (feed/permeate)).

To evaluate the pervaporation performance, the membrane flux and water concentration in the permeate were determined (Fig. 12). In all cases, very low amounts of permeate were formed and the permeate contained mainly water, thus a highly accurate estimation of other components was not feasible. Since the amounts of water formed in the system were very small, the fluxes of the permeate were very low as well, with less than 0.5 ml of permeate collected after 15 h. This phenomenon can be supported by two contributing factors: first, the diminished yield observed for the heterogeneous catalytic system, which lead to the formation of minimal amounts of water and, second, the less pronounced water permeation through the non-swollen, coated membranes, which was in line with the initial pervaporation tests. One of the possible options to overcome the poor membrane flux would be *via* increasing the reaction temperature. Based on the findings of the pre-studies on pervaporation, it was observed that increasing the temperature enhanced the flux. However, with the set-up available, the highest safe operation temperature could not exceed 95 °C. This leaves an outlook for future experiments with a remodelled membrane reactor.

## Conclusions

In this work, catalytically active, composite flat-sheet membranes coated with mixtures of PVA and ILs were fabricated and used for the esterification between *n*-butanol and acetic acid, coupled with simultaneous water removal *via* pervaporation.

The composite flat-sheet membranes were functionalized with a catalytic layer containing Brønsted-acidic imidazolium-based IL grafted to PVA. The composites were produced by dip-coating commercial pervaporation membranes, resulting in a thin (12 μm thick), defect-free catalytic layer formed on top of the pervaporation membrane support. The penetration depth of the ILs, determined with the help of LA-ICP-MS, indicated their presence in the upper part of the coating, thus enabling their effective participation in esterification as catalysts.

To determine the impact of the IL anion, two different ILs, with the same imidazolium-based cation but different, namely sulfate and bromide, anions were compared. The coated membranes showed enhanced water separation factors (reaching 209.5 [−] for the IL with the bromide anion and 178.7 [−] for the sulfate-based IL) compared to the unfunctionalized membrane (82.5 [−]), which could be explained by the reduced swelling and lower wettability of the coated membranes. As shown in catalytic studies, the composite membranes formed with both ILs could promote the formation of *n*-butyl acetate, and the IL with the [HSO_4_]^−^ anion catalyzed the esterification more effectively than the one with [Br]^−^. However, the positive effect of water removal *via* catalytic composite membrane pervaporation was less explicit, as full conversion was not achieved. Future research will focus on redesigning the catalytic membrane reactor and examining the impact of different acetic acid to *n*-butanol ratios.

## Data availability

The data supporting this article (including Technical Data Sheet of membranes; procedures of ILs' synthesis; membrane performance calculations and ^1^H NMR and FT-IR spectra of coating solutions and membrane composites) have been included as part of the ESI,† are available and free of charge.

## Author contributions

The manuscript was written through contributions of all authors. All authors have given approval to the final version of the manuscript.

## Conflicts of interest

There are no conflicts to declare.

## Supplementary Material

RE-010-D4RE00388H-s001
