# Supplementary material for: Acid-functionalized PVA composite membranes for pervaporation-assisted esterification
Source: React Chem Eng. 2024 Nov 15;10(2):360–70. doi: 10.1039/d4re00388h (PMC11600398; doi:10.1039/d4re00388h)
Supplement: RE-010-D4RE00388H-s001 [file RE-010-D4RE00388H-s001.pdf]

Supplementary Information (SI) for Reaction Chemistry & Engineering.
This journal is © The Royal Society of Chemistry 2024

Supplementary Information

Acid-Functionalized PVA Composite Membranes for Pervaporation-Assisted Esterification

Julia Piotrowska,[a] Christian Jordan,[b] Kristof Stagel,[a] Marco Annerl,[c] Jakob Willner,[c] Andreas Limbeck,[c] Michael Harasek[b] and Katharina Bica-Schröder*[a]

[a.] *TU Wien, Institute for Applied Synthetic Chemistry, Getreidemarkt 9/E163, Austria.*
[b.] *TU Wien, Institute of Chemical, Environmental and Bioscience Engineering, Getreidemarkt 9/E166, Austria.*
[c.] *TU Wien, Institute of Chemical Technologies and Analytics, Getreidemarkt 9/E164, Austria*

Corresponding Authors

* Katharina Bica-Schröder, phone: +43 1 58801 163601,
mail: katharina.schroeder@tuwien.ac.at

Number of pages: 7

Number of tables: 1

Number of figures: 3

Number of equations: 4

1. Technical Data Sheet of commercial pervaporation membranes

Table S1 Technical Data Sheet of pervaporation membranes, provided by DeltaMem AG

**Membrane type**	PERVAP^{TM} 4100
**Typical application**	Standard membrane, developed for most dehydration of volatile organic mixtures
**Feed temperature**	Max. short term operating temperature – 105 ^{o}C
**Feed pressure**	Above the feed vapor pressure, typically up to 4 bar
**Melting point**	Typical operating pH range: 5-8, operation outside these pH values – acceptable in some cases
**Compatibility with chemicals**	Fully compatible with: alcohols, ether (including cyclic ethers), acetates / esters, ketones, hydrocarbons, acetonitrile<br><br>Conditionally compatible with: aldehydes and derivatives <30ppm (as acetaldehyde), organic acids <0.1 % w/w, acetals / ketals, special solvents (DMF, DMSO, NMP, DMAc <0.1 % w/w<br><br>Not compatible with amines (e.g. MMA) <500 ppm, mineral acids and peroxides

2. General procedure for the batch-wise synthesis of ionic liquids

Both ionic liquids were synthesized based on the procedure described by Liu et. al.[1]

(1) Liu, L. K.; Deng, J. H.; Guo, Y. M. Synthesis of Coumarin Derivatives in a Microfluidic Flow System Employing the Pechmann Condensation: A Case Study. *J. Chinese Chem. Soc.* **2020**, *67* (12), 2208–2215. https://doi.org/10.1002/jccs.202000371.

Synthesis procedure for 3-(4-sulfonyl)-1-vinyl-imidazolium hydrogen sulfate (IL1) and of 3-(4-sulfonyl)-1-vinyl-imidazolium bromide (IL2)

1-vinylimidazole (0.12 mol) and 1, 4-butanesultone (0.12 mol) were mixed in a 250 mL round bottom flask and dissolved in 60 mL of acetonitrile. The mixture was stirred at 42-45°C for 16 h. The solvent was removed, and white solid zwitterion was washed repeatedly with ether to remove non-ionic residues, filtrated through a Buchner funnel and dried in vacuum for 4h. A stoichiometric amount (0.12 mol) of HSO_4 (for IL1 synthesis) or HBr (for IL2 synthesis) was added dropwise, the mixture was stirred for 6h at 80°C. The viscous liquid was washed three times with ether and dried in vacuum to form IL-1.

3. Calculations of membrane pervaporation performance

To evaluate the membrane separation performance, the two main parameters were considered: membrane flux J, partial flux of component *i* and separation factor α_i. They can be described with Equations (S1-S3):

$$J = \frac{m}{A \cdot t} \tag{S1}$$

Where J is membrane flux [g·m^{-2}·h^{-1}], m stands for the mass of collected permeate [g], t is pervaporation time [h] and A stands for the effective area of a membrane (0.006793 m^2). For component *i*, partial flux J_i is described by the following equation:

$$J_i = J \times \omega_i^P \tag{S2}$$

where ω_i^P is the weight fraction of component i in permeate.

Separation factor αi for a given compound i can be defined as:

$$\alpha_i = \frac{\omega_i^P/(1-\omega_i^P)}{\omega_i^F/(1-\omega_i^F)} \tag{S3}$$

Where ω_i^P stands for weight fraction of the component i in permeate and ω_i^F is weight fraction of the compound *i* in feed.

The enrichment factor β_i, calculated from Equation (S4) indicates the degree to which component i (of greater permeability), is enriched.

$$\beta_i = \frac{\omega_i^P}{\omega_i^F} \tag{S4}$$

4. Full ^{1}H NMR spectra of IL1, IL2 and neat PVA

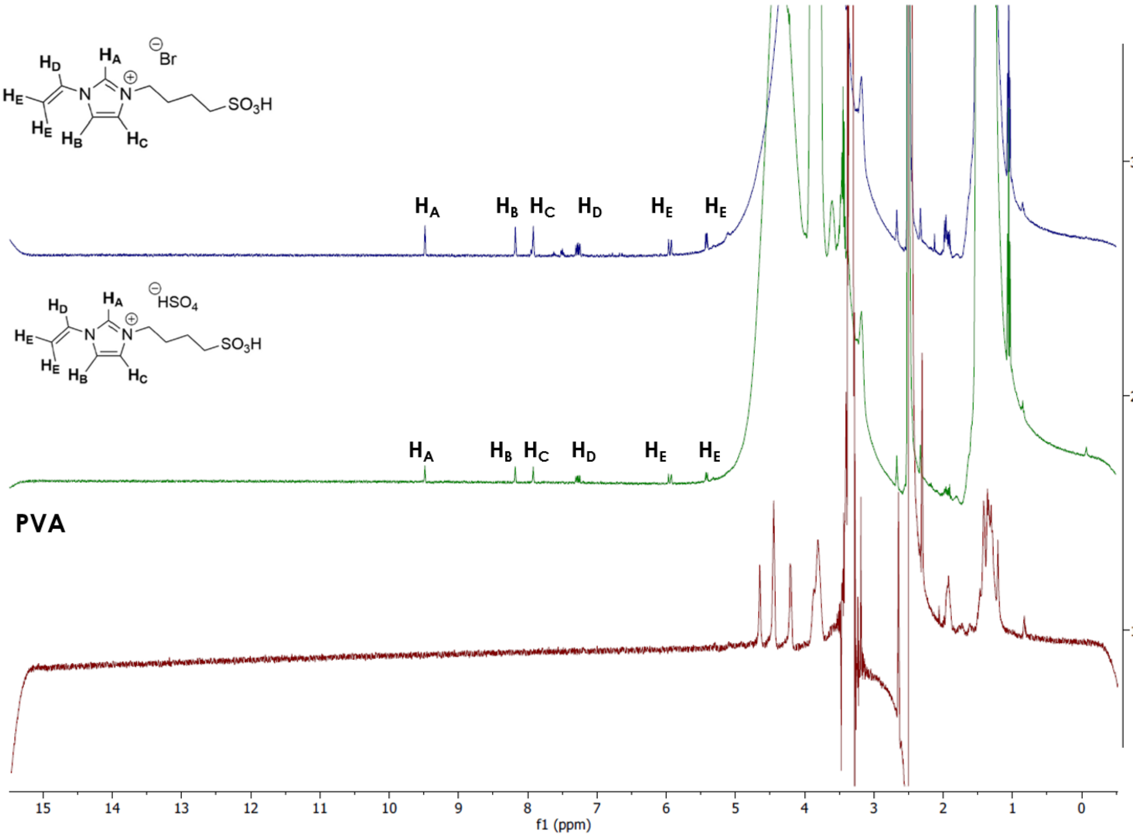

Fig. S1 Full ^{1}H NMR spectra of PVA/IL1(top), PVA/IL2 (middle) and neat PVA (bottom)

The assignment of signals characteristic for ILs, according to the markers in Fig. S1 is as follows: 9.49 (1H, s, HA), 8.19 (1H, s, HB), 7.93 (1H, s, HC), 7.31 (1H, m, HD), 5.93 (1H, m, HE), 4.63 (1H, m, HE).

5. FT-IR spectra of coated membranes before and after pervaporation test

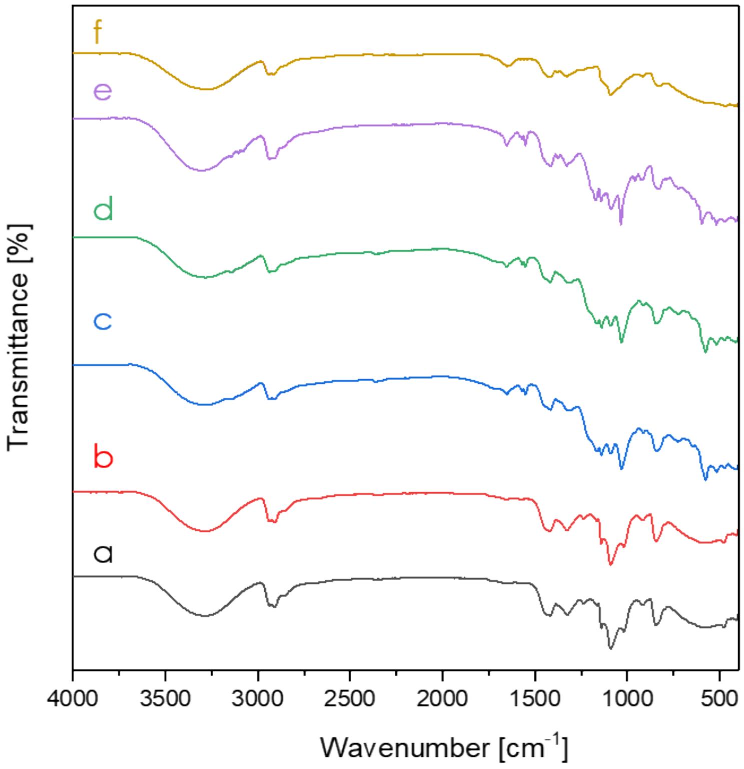

Fig. S2 FT-IR spectra of composite membranes, coated with PVA (a,b), PVA/IL1 (c,d), PVA/IL2 (e,f) before (a,c,e) and after (b,d,f) pervaporation test.

6. ^{1}H NMR spectra of the supernatant after catalyst leaching tests

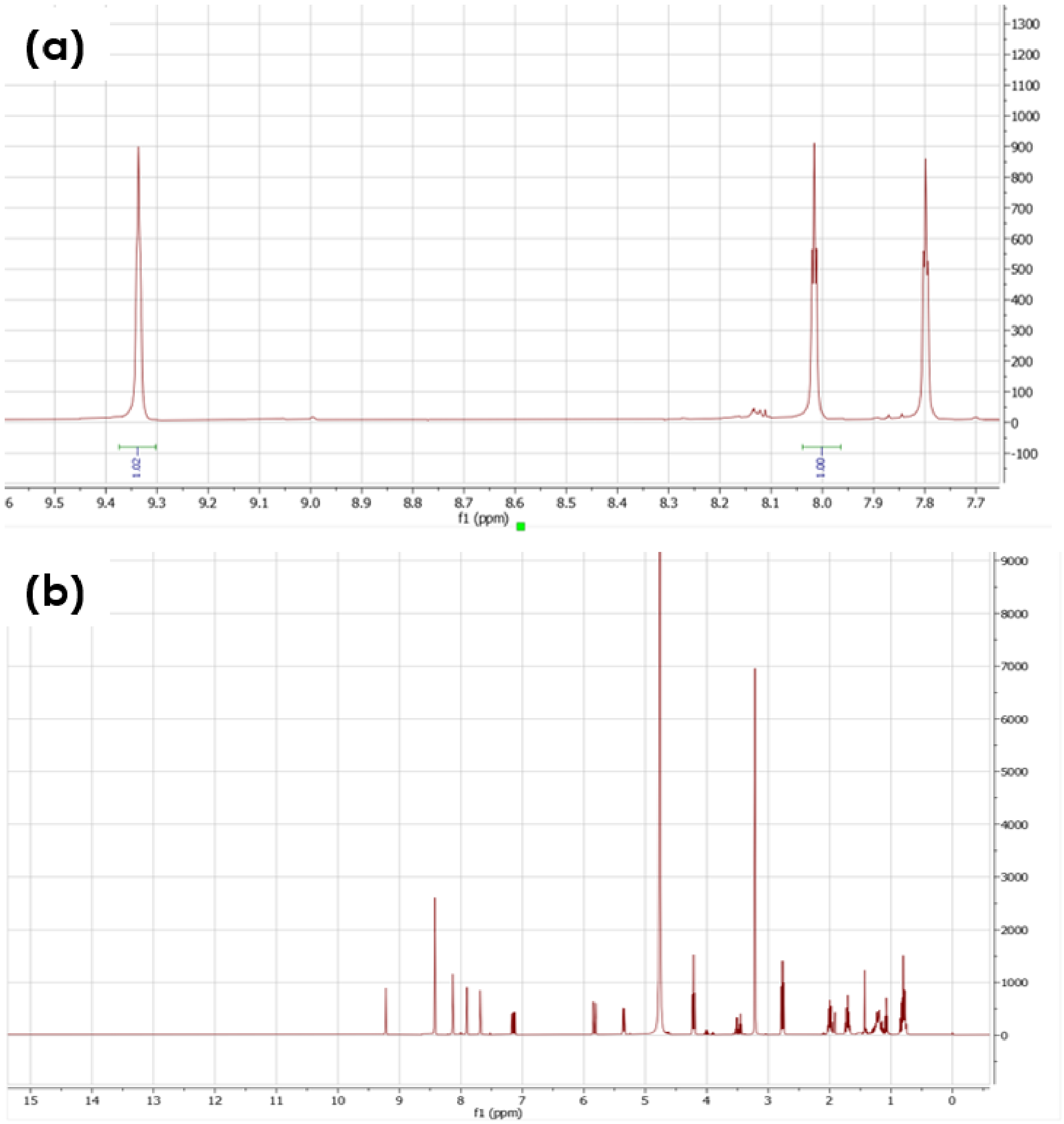

Fig. S3 ^{1}H NMR spectra of supernatant after the leaching tests with PVA/IL1-coated (a) and PVA/IL2-coated (b) membranes.
